# *In-vivo* analysis of epicutaneous pressure distribution beneath a femoral tourniquet – an observational study

**DOI:** 10.1186/s12891-015-0454-0

**Published:** 2015-01-31

**Authors:** Klaus Edgar Roth, Boris Mandryka, Gerrit Steffen Maier, Uwe Maus, Manfred Berres, Jan-Dirk Rompe, Friedrich Bodem

**Affiliations:** Department of Orthopedics and Traumatology, Universitätsmedizin Mainz, Langenbeckstrasse 1, 55131 Mainz, Germany; Institut für Medizinische Biometrie, Epidemiologie und Informatik, Unimedizin Mainz und RheinAhrCampus der Hochschule Koblenz, Mainz, Germany; OrthoTrauma Evaluation Institute, Mainz, Germany

## Abstract

**Background:**

Compression of the tissue beneath tourniquets used in limb surgery is associated with varying degrees of soft tissue damage. The interaction between fluids and applied pressure seems to play an important role in the appearance of skin lesions. The extent of the transfer of force between the tourniquet and the skin, however, has yet to be studied. The aim of the present study was to quantify *in-vivo* the transfer of pressure between a tourniquet and the skin of the thigh.

**Methods:**

Pressure under the tourniquet was measured using sensors in 25 consecutive patients over the course of elective surgical procedures. Linear mixed modeling was used to assess the homogeneity of the distribution of pressure around the circumference of the limb, variation in pressure values over time, and the influence of limb circumference and the Body-Mass-Index (BMI) on pressure transfer.

**Results:**

Mean pressure on the skin was significantly lower than the inner pressure of the cuff (5.95%, 20.5 ± 9.36 mmHg, p < 0.01). There was a discrete, but significant (p < 0.001) increase in pressure within the first twenty minutes after inflation. Sensors located in the area of overlap of the cuff registered significantly higher pressure values (p < 0.01). BMI and leg circumference had no influence on the transfer of pressure to the surface of the skin (p = 0.88 and p = 0.51).

**Conclusions:**

Pressure transfer around the circumference of the limb was distributed inhomogeneously. The measurement series revealed a global pressure drop compared to the initial pressure of the cuff. No relationship could be demonstrated between the pressure transferred to the skin and the BMI or limb circumference.

## Background

Controlled suppression of circulating blood volume by means of an exsanguination cuff operating through suprasystolic compression during limb surgery leads to improved clarity at the operation site and minimizes intraoperative blood loss [1,2]. The surgeon is able to achieve higher precision in a blood-free setting [2], which is beneficial to the patient’s safety. It is, however, also the case that an inflated exsanguination cuff leads to blood pooling [3] at the height of and distal to the affected section of the limb. This has widespread systemic-metabolic consequences [4,5] and can lead to decompensation in patients with a low cardiopulmonary reserve [6,7].

Local complications such as nerve lesions [8] or muscular and vascular damage [9] that might occur in the context of pressurization at deeper tissue levels appear to arise from a convergence of compression and locally disturbed perfusion [10]. Skin conditions such as bruising, blistering, and hematoma formation, or even burns or necrosis, are less commonly seen (incidence 0.04% to 1.5% [11,12]), and their exact pathogenesis has been differently interpreted [13,14]. Both the application of excessive cuff pressure and prolonged usage of an improperly placed pressure cuff [11,12], and thermo-mechanical/chemical causes [15,16] have been considered. The interaction between fluids and applied pressure would seem to play an important role in the appearance of skin lesions [15], particularly when the initial placement of the pressure cuff was too loose [12].

In general the value for internal pressure of the cuff given by the manometer is, based on an idealized hydrostatic-physical model, equated to the actual mechanical pressure experienced at the surface of the skin, although this has yet to be validated through *in-vivo* studies. The extent to which pressure differences exist in different areas around the circumference of the limb, for example at sites where pressure cuff segments overlap, remains unclear. The degree of overlap, however, is strongly dependent on limb circumference. The possibility that the mechanics of pressure transfer differ in this area from that in the rest of the pressure cuff means that changes in local contact pressure can not be completely excluded as possible triggers for pressure-related complications.

In view of this the aim of this study was a location dependent investigation of pressure transfer to the upper surface of the extremity under the influence of intrinsic factors.

## Methods

All procedures used in the present study had been approved by the ethics committee of the state medical association of Rhineland-Pfalz, Mainz, Germany. After full explanation of the procedures all participants gave their signed consent to the study.

The study was performed on a collective of 25 consecutive patients who underwent surgical intervention distal to a pressure cuff placed on the thigh. Demographic data, height, weight, BMI, and the circumference of the thigh 20 cm above the knee joint were collected before the operation. Intraoperative blood pressure was obtained from the anesthesia records. Undesirable side-effects identified postoperatively were noted and analyzed with respect to pressure conditions obtained intraoperatively. Reasons for exclusion from the study were skin diseases, arterial circulatory disorders, leg circumference of less than 48 cm, or a BMI > 35 kg/cm^2^.

All measurements were obtained using the same one-chambered ischemic pressure cuff with a size of 110 × 800 mm and a pneumatic compression system and Velcro fastener (Ulrich medical®, Ulm, Germany). Occlusion pressure was registered using a miniature mobile tourniquet, balbina™, operating over a pressure range of 30-650 mmHg.

**Figure 1:**
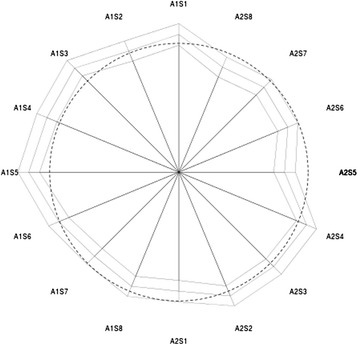
**Star plot of fitted means at time 0 (middle polygon) with lower and upper 95% confidence limits (inner and outer polygon).** Dashed circle shows overall mean. Schematic diagram of the sensor layout; A = Array, S = Sensor. Sensors located in the area of overlap of the cuff (A1S1-3) showed significantly higher pressure values than the anteromedially positioned sensor A2S8.

Pressure transduction was performed by sensors of the type FSR-174 (Force-Sensing-Resistor; International Electronics & Engineering-IEE®,Findel, Luxembourg). These had a diameter of 27.8 mm. 16 sensors were placed in standardized positions around the limb (Figure 1). The pressure acting on these sensors altered their electrical resistance and the signals so generated were transmitted to a measuring system outside of the operating area. Measurements were obtained every 3 seconds and continuously saved and visually monitored. Subsequent processing and analysis of the data was performed by means of a spreadsheet (Excel 2007, Microsoft Corp., Redmond, USA) and with R [17]. For each patient the first 60 seconds and the last 10 values (or 30 seconds) of measurement were deleted in order to restrict measurement to the period in which full inflation was maintained. Preliminary testing was performed to calibrate the individual sensors and to analyze their creep behavior.

**Figure 2:**
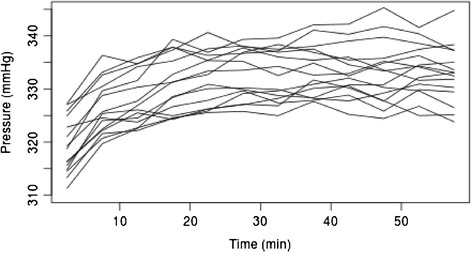
**Means per position, aggregated over patients and intervals of 5 minutes.**

In order to maintain the pressure detectors at defined positions on the limb the sensors were placed in pockets sown onto the internal surface of the pressure cuff. The 16 sensors were dispersed in 2 pressure-monitoring arrays, each consisting of 8 coupled sensors. Sensor 1 of the 1^st^ array was always placed ventromedially on the thigh. The remaining 7 sensors of Array 1 were then spread out successively from lateral to dorsal. The 1^st^ sensor of the 2^nd^ array was then placed dorsomedially, with the remaining 7 sensors proceeding from medial to ventral (Figure 2). Thus a comparable layout of measurement points was obtained independent of the limb involved.

Each measurement was performed with two layers of cotton padding. A standardized procedure was followed, with constant room temperature and sustained control of the measurement environment. A postoperative examination of the skin under the pressure cuff was carried out in dry and disinfectant free surroundings.

Incision of the skin was preceded by mechanical exsanguination using elastic ligatures. Subsequently the tourniquet was inflated to a manometer pressure of 350 mmHg [18] with a maximum application of 2 hours [3,19]. Recording of data began after inflation and ended after deflation of the pressure cuff. As the sensors showed unequal variations in resistance during inflation and deflation, and a degree of short-term inaction was apparent during the process of inflation, the first minute of data and the last 10 measurements were excluded from statistical analysis. Implausible measurements, arising, for example, from repositioning movements during the operation, were interpolated with the mean of the previous 20 measurements.

### Statistics

Statistical analysis was performed using SPSS Version 17.0 und R. An overview of the mean for each sensor position was obtained, along with the associated minima, maxima and standard deviation. Data were analyzed in a linear mixed effects model with random effects for patients. This accounts for inter-correlations of data values within patients. A plot of mean pressure over time suggested the application of an asymptotic model for time of the type *y = a – be*^*-ct*^ with *a,b,c >* 0*.* The parameter *c* was estimated beforehand in a non-linear fixed effects model. The transformed covariable *x = e*^*-ct*^ and the positions on the device were included as fixed effects in the mixed effects model. Interactions of positions with time could not be included in the model due to numerical problems in the algorithm. However, separate models for each position were estimated. Model assumptions were checked by graphical analysis of residuals. BMI, limb circumference and duration of application of the device were tested for inclusion in the mixed model. Average pressure per patient was correlated with demographic variables.

## Results

No undesired local or systematic effects were observed during application of the tourniquet or postoperatively in any of the patients. All 25 patients could be included in the final analysis. Descriptive statistics for the subjects (BMI, height, weight, thigh circumference, systolic blood pressure) are given in Table 1. The average age of the 14 female and 11 male patients was 53.7 years (range 23-78). The surgical procedures used for the 25 patients consisted of 6 knee prostheses, 7 knee arthroscopies, 6 open surgical interventions at the knee joint, and 6 interventions to the foot.

**Table 1:** **Descriptive statistics of the patients (Min.: Minimum, Max.: Maximum, SD: Standard deviation, Qu.: Quartile, BMI: Body-Mass-Index)**

**Quantile**	**Height (cm)**	**Weight (kg)**	**BMI (kg/cm** ^2^ **)**	**Thigh circumference (cm)**	**RR Syst. (mmHg)**
Min	153	52	20.83	48	120
1st Qu.	167	77	24.91	54	130
Median	174	81	26.2	56	140
Mean	173.2	85.32	28.42	57.32	138.8
3rd Qu.	179	92	30.35	60	146
Max.	195	146	41.75	69	174
SD	9.57	18.12	5.26	5.19	12.6

Combination of all measurement series (P_1-25) revealed a global pressure drop of 20.5 ± 9.36 mmHg (*p* ≤ 0.01) or 5.95 ± 3.11% compared to the initial pressure of the cuff (350 mmHg). As may be seen in Table 2, the lowest mean value was seen at sensor A2S5 (323.6 mmHg), while the highest pressure was recorded by sensor A1S5 (339.5 mmHg).

**Table 2:** **Univariate statistics for each sensor position (Qu.: Quartile, Min.: Minimum, Max.: Maximum)**

**Sensor position**	**Min.**	**1st Qu.**	**Median**	**Mean**	**3rd Qu.**	**Max.**
A1S1	238.8	323.6	336.3	333.8	344.8	421.1
A1S2	232.4	317.2	335.2	331.5	346.9	413.7
A1S3	255.7	325.7	337.3	336.9	349.0	412.6
A1S4	244.1	321,4	337,3	335,4	347,9	419,0
A1S5	173.0	326,7	339,5	337,9	347,9	427,4
A1S6	227.1	318,3	334,2	331,0	344,8	399,9
A1S7	238.8	314,0	325,7	327,4	343,7	402,0
A1S8	233.5	317,2	329,9	329,0	342,6	388,2
A2S1	79.76	317,2	327,8	327,4	338,4	408,4
A2S2	88.24	320,4	332,0	332,5	343,7	399,9
A2S3	63.86	320,4	334,2	332,4	344,8	421,1
A2S4	141.2	320,4	334,2	333,8	346,9	394,6
A2S5	144.4	308,7	323,6	322,8	337,3	396,7
A2S6	93.54	315,1	328,3	326,9	340,5	388,2
A2S7	111.6	317,2	331,0	327,7	340,5	376,6
A2S8	123.3	314,0	327,8	325,6	340,5	382,9

In the non-linear model the coefficient *c* was estimated as *c =* 0.161. The fixed effects model for sensor position A1S1 was Pressure = 336.1 – 23.02 *e*^*-*0.161*t*^*,* 1 ≤ *t* ≤ 60 (minutes). For other sensor positions the coefficient 336.1 had to be replaced by the respective coefficient from Table 3.

**Table 3:** **Coefficients and confidence limits of the mixed model’s fixed effects**

**Sensor position**	**Value**	**Std. error**	**Lower 95% CI**	**Upper 95% CI**
A1S1	**336.12**	**1.87**	**332.45**	**339.79**
A1S2	**333.86**	**1.87**	**330.18**	**337.53**
A1S3	**339.30**	**1.87**	**335.63**	**342.97**
A1S4	**337.74**	**1.87**	**334.07**	**341.42**
A1S5	**340.21**	**1.87**	**336.54**	**343.89**
A1S6	**333.38**	**1.87**	**329.71**	**337.05**
A1S7	**329.76**	**1.87**	**326.08**	**333.43**
A1S8	**331.32**	**1.87**	**327.65**	**334.10**
A2S1	**329.80**	**1.87**	**326.12**	**333.47**
A2S2	**334.85**	**1.87**	**331.18**	**338.52**
A2S3	**334.73**	**1.87**	**331.06**	**338.40**
A2S4	**336.13**	**1.87**	**332.45**	**339.80**
A2S5	**325.13**	**1.87**	**321.46**	**328.81**
A2S6	**329.29**	**1.87**	**325.61**	**332.96**
A2S7	**330.10**	**1.87**	**326.43**	**333.77**
A2S8	**327.96**	**1.87**	**324.28**	**331.63**
e ^-0,161*t*^	**-23.02**	**0.150**	**-23.31**	**-22.73**

The model was specified without intercept: coefficients of sensor positions are estimated means. The standard error of all estimated means is 1.87. (CI: Confidence interval).

Between patient variability (SD of random effects) was 9.4 mmHg und standard deviation of the residuals (i.e. variability not explained by position, time and patient) was 17.9 mmHg. Residuals of the model had some 20 outliers on the left. When these were removed, a quantile plot of the residuals showed no deviation from normality and the parameters of the model remained almost unchanged. Overall the confidence intervals shown in Table 3 and Figure 2 reveal an inhomogeneous pressure distribution over the whole circumference of the limb.

**Figure 3:**
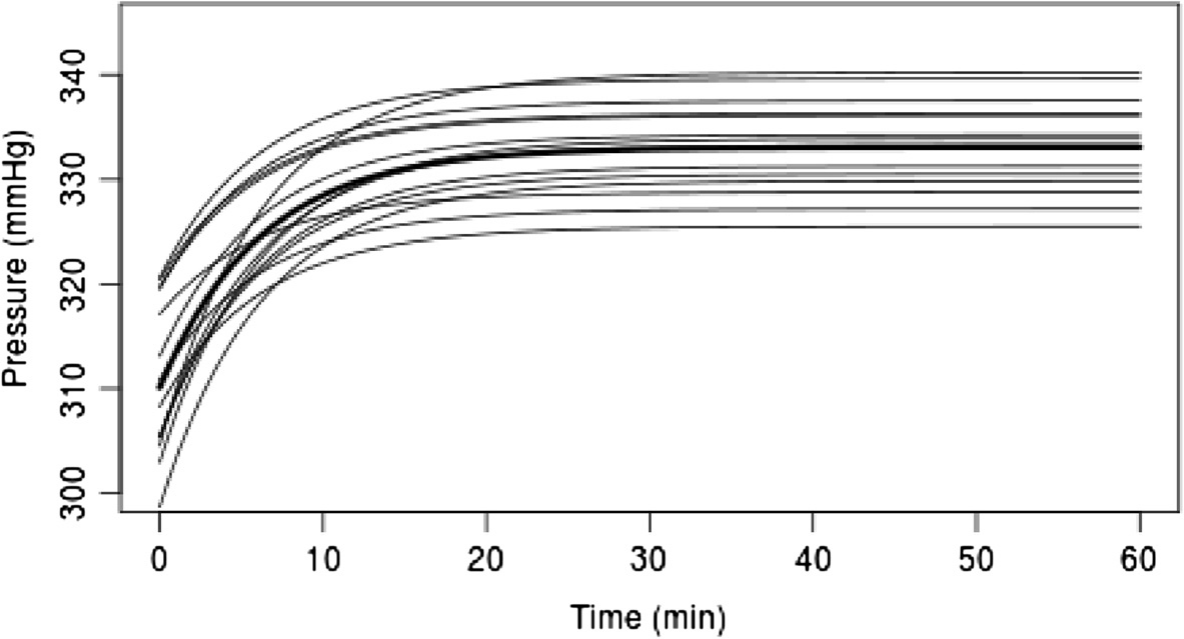
**Fitted asymptotic curves from separate mixed models for each position.** Thick line shows asymptotic curve fitted for all positions simultaneously.

Over the course of time there was a slight (but significant, p < 0.001) increase in pressure, on average by 20 mmHg within the first 20 minutes (Figure 3, Figure 1 thick line). Increasing pressure was also seen when each position was analyzed separately (Figure 3, Figure 1 thin lines).

Inclusion of other potential predictors in the model did not improve the model: p-values of regression coefficients were 0.88 (BMI), 0.51 (limb circumference) and 0.37 (duration of application). The pressure measurements obtained were independent of the type of operation (prosthesis, arthroscopy, etc.) involved.

The degree to which cuff pressure exceeded systolic blood pressure, which is an important parameter for the safe use of the pressure cuff was, on average, 193.51 ± 10.91 mmHg (*p* ≤ 0.01) and at no point fell below 188.2 ± 21.7 mmHg. In connection to this we should mention the intraoperative bleeding experienced by patient 15, which was not explainable by surgical procedures and led to deflation of the pressure cuff before completion of the surgical intervention. Analysis of the pressure sensor data of this patient failed to reveal the drop in pressure suspected by the surgeons, but instead indicated an inconspicuous course.

## Discussion

The use of tourniquets in surgery of the extremities is not without controversy. Local and systemic incidents represent risk factors that can increase postoperative morbidity and mortality [7,13]. With regard to these dangers Odinsson [16] came to the conclusion that the present rate of complications, despite constructive improvements of the pressure cuffs, is no different from that seen in the seventies.

Up until now modifications in pressure cuff design have largely concerned homogenization of the compressive force in target tissue [14,16,20], as peaks of pressure are seen as important factors in tissue traumatization [10]. A few *ex-vivo* studies [21-23] have been able to show that a decrease in pressure occurs as it moves deeper into the tissue. The present study is, to the authors’ knowledge, the first *in-vivo* study of the pressure conditions beneath a tourniquet. The results of the present study show that a reduction in pressure is already apparent during the pressure transfer between the tourniquet and the patient’s skin (on average 5.95%). We would attribute this loss of pressure to the incomplete hydrostatic properties of the cushioning material. The closed, approximately plain cylindrical structure of the cuff cushioning, in contrast to the expected behavior of a water cushion, initiates a partially elastic compression of the cushioning material in a tangential direction. In this way the pressure cuff force acting on all sides perpendicularly in the padding also exerts lateral force components. Through this effect, which increases with the thickness of the cushioning layer, the transfer of the perpendicular pressure cuff force to the surface of the skin is reduced. Thus it may be assumed that each layer of the cushioning diverts a portion of the exerted pressure, which is thus no longer passed on to the succeeding layer. In this way the protective effect of increasing thickness of cushioning against cutaneous lesions described by some authors [11,22] can be attributed to these mechanical properties.

It is interesting to note that this initial loss in pressure transfer was followed in all test data by a slowly increase in the pressure curve up to a plateau level (Figure 1). We are unable to explain this phenomenon, but may be suspected that the elastic material properties of the centre of the cuff play a causal role in this.

The intraoperative bleeding seen in patient 15 could not be attributed to malfunction of the cuff. In the literature such problems have been described in patients with massive calcification of the arterial wall or Monckeberg’s arteriosclerosis [23,24]. In advanced stages of calcification of the arterial wall cuff pressure is insufficient to achieve compression of the arterial vascular tree. The average pressure difference of 193.5 ± 12.91 mmHg above medium systolic pressure and the stable pressure curves seen in all data sets indicate strongly the reliability of the pressure cuff applied here. Comparison of our measurements with previous recommendations of cuff pressure levels in the literature [18,25] led us to the conclusion that our output pressure of 350 mmHg was unnecessarily high. On the basis of our results we will in future reduce our output and hope thereby to achieve a further reduction in the risk of tourniquet-associated complications.

As a result of the very large amount of data, highly significant pressure differences were observed between sectors used in the present study (Figure 3). Sensors located in the area of overlap of the pressure cuff (A1S1-3) showed significantly higher pressure values than the anteromedially positioned sensor A2S8, although the absolute pressure differences only lay between 6 to 11 mmHg. Although the absolute pressure differences were not very marked, the differences in tension could be considered to contribute a risk for a skin lesion. If this observation is carried over to the clinical situation it means that, in the course of a harmonious pressure distribution of the compression ratio, the area of overlap should be as narrow as possible. This indicates that persons with a smaller leg circumference need shorter cuffs than those with a large circumference. For clarification of this interpretation of the data the authors are planning to carry out further experimental analysis.

We were unable to observe the inverse correlation between compression ratios and limb circumference reported in the literature [26], although in the latter measurements were made deep in the tissue, rather than at the surface of the skin. The finding that the parameters BMI and leg circumference had no influence on the transfer of pressure to the surface of the skin is not in contradiction with other published data describing a loss of pressure at deeper tissue levels, especially as the measurements presented here are restricted to the level of the skin.

All assessment of the results must bear in mind the physical properties of the sensors. Pressure peaks caused by positioning of the limb during the operation are included in the measurements and could affect the results presented.

## Conclusions

There was an inhomogenous transfer of pressure around the circumference of the limb. In accordance with earlier *ex-vivo* studies we found a significant loss of pressure in the transfer of force between the tourniquet and the skin *in-vivo*. In contrast to the conditions in deeper laying tissue, however, this loss is not influenced by the circumference of the extremity.
